# Deep Learning-Assisted Diagnostic System: Implant Brand Detection Using Improved IB-YOLOv10 in Periapical Radiographs

**DOI:** 10.3390/diagnostics15101194

**Published:** 2025-05-08

**Authors:** Yuan-Jin Lin, Shih-Lun Chen, Ya-Cheng Lu, Xu-Ming Lin, Yi-Cheng Mao, Ming-Yi Chen, Chao-Shun Yang, Tsung-Yi Chen, Kuo-Chen Li, Wei-Chen Tu, Patricia Angela R. Abu, Chiung-An Chen

**Affiliations:** 1 Department of Program on Semiconductor Manufacturing Technology (PSMT), Academy of Innovative Semiconductor and Sustainable Manufacturing (AISSM), National Cheng Kung University, Tainan City 701401, Taiwan; m28121562@gs.ncku.edu.tw; 2 Department of Electronic Engineering, Chung Yuan Christian University, Taoyuan City 320317, Taiwan; chrischen@cycu.edu.tw (S.-L.C.); s11126213@cycu.edu.tw (Y.-C.L.); s11126215@cycu.edu.tw (X.-M.L.); 3 Department of Operative Dentistry, Taoyuan Chang Gung Memorial Hospital, Taoyuan City 33305, Taiwan; louiszzzzz@cgmh.org.tw; 4 Department of Family Dentistry, Taoyuan Chang Gung Memorial Hospital, Taoyuan City 33305, Taiwan; terresa422@cgmh.org.tw; 5 Department of Electrical Engineering, Ming Chi University of Technology, New Taipei City 243303, Taiwan; joannechen@mail.mcut.edu.tw; 6 Department of Electronic Engineering, Feng Chia University, Taichung City 40724, Taiwan; tsungychen@fcu.edu.tw; 7 Department of Information Management, Chung Yuan Christian University, Taoyuan City 320317, Taiwan; 8 Department of Electrical Engineering, National Cheng Kung University, Tainan City 701401, Taiwan; wctu@gs.ncku.edu.tw; 9 Ateneo Laboratory for Intelligent Visual Environments, Department of Information Systems and Computer Science, Ateneo de Manila University, Quezon City 1108, Philippines; pabu@ateneo.edu

**Keywords:** clinical decision support systems, implant brand, image enhancement, dental medical diagnosis, periapical radiographs, you only look once

## Abstract

**Background and Objectives**: Implant brand identification is critical in modern dental clinical diagnostics. With the increasing variety of implant brands and the difficulty of accurate identification in periapical radiographs, there is a growing demand for automated solutions. This study aims to leverage deep learning techniques to assist in dental implant classification, providing dentists with an efficient and reliable tool for implant brand detection. **Methods**: We proposed an innovative implant brand feature extraction method with multiple image enhancement techniques to improve implant visibility and classification accuracy. Additionally, we introduced a PA resolution enhancement technique that utilizes Dark Channel Prior and Lanczos interpolation for image resolution upscaling. **Results**: We evaluated the performance differences among various YOLO models for implant brand detection. Additionally, we analyzed the impact of implant brand feature extraction and PA resolution enhancement techniques on YOLO’s detection accuracy. Our results show that IB-YOLOv10 achieves a 17.8% accuracy improvement when incorporating these enhancement techniques compared to IB-YOLOv10 without enhancements. In real-world clinical applications, IB-YOLOv10 can classify implant brands in just 6.47 ms per PA, significantly reducing diagnostic time. Compared to existing studies, our model improves implant detection accuracy by 2.3%, achieving an overall classification accuracy of 94.5%. **Conclusions**: The findings of this study demonstrate that IB-YOLOv10 effectively reduces the diagnostic burden on dentists while providing a fast and reliable implant brand detection solution, improves clinical efficiency, and establishes a robust deep learning approach for automated implant detection in PA.

## 1. Introduction

Oral health is essential to overall health, contributing to psychological and physiological well-being. Good oral health enhances an individual’s quality of life; however, it is well known that tooth loss negatively impacts occlusal force, hindering proper mastication [1]. Additionally, missing teeth may affect employment opportunities [2] and compromise overall health [3,4]. In recent years, there has been a growing awareness of the importance of oral health, leading more individuals to seek dental treatment for tooth restoration and to improve their daily quality of life [5]. Furthermore, dental implant treatment has become a widely accepted and proactive approach for tooth replacement, indicating that implant therapy is an essential trend in modern dentistry [6,7]. In implant therapy, periapical radiographs (PAs) are commonly used for implant assessment because they provide detailed visualization of the implant site and surrounding structures [8,9]. PA offers detailed images of individual teeth, typically capturing two to three teeth per image, including their roots and surrounding bone structures. The high resolution allows for the precise monitoring of bone changes around implants, aiding in the early detection of potential complications.

**Figure 1:**
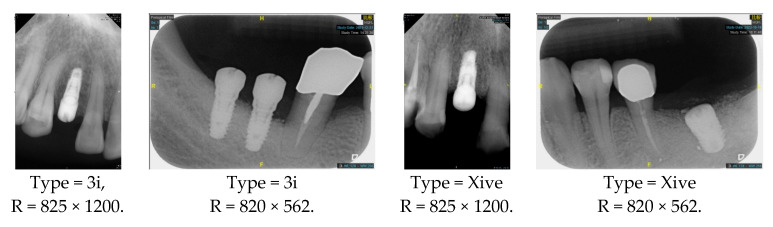
Imaging of two implant brands at different resolutions.

However, since the implant launched onto the market, dental treatment related to implant issues has become the everyday practice of dentists’ lives in clinics [10]. The design of the implants, from shape to surface coating, is different from brand to brand [11]. But it is inevitable to come into implant complications [12,13], such as screw fracture, healing abutment loss, etc., whether the clinician itself treats the implant or implant prosthesis. Different surface coatings [14,15] and threads of the implant design [16,17] may be easily identified in vitro. Moreover, once the implants are in vivo, the clinicians can only identify implant brands through radiography or the clinicians’ familiarity with the implant. If the clinicians are not familiar with the brands, then it is hard to tell which brand the implant is from in vivo. As the number of implant brands on the global market grows, it has become increasingly complex for clinicians to identify and familiarize themselves with all available implant systems accurately. 3i and Xive are two of the most widely used and clinically validated implant brands worldwide, with excellent long-term success rates [18] and reliable implant-abutment connection designs [19]. Therefore, we strategically focused on 3i and Xive implants in this study to ensure clinical relevance and practical significance. Concentrating on these two brands allows our model to address many real-world clinical cases and enhances its applicability in everyday dental practice. Clinicians will likely encounter both implant brands due to their widespread use in global dental clinics [20]. Moreover, as the implant’s screwdrivers differ from brand to brand, the identification of implant brands is very important to clinicians and patients. The marginal accuracy of the implant-abutment connection may vary due to misidentification and lead to unknown clinical problems [21,22]. The PA imaging results of two implant brands of different types at different resolutions (R) are shown in Figure 1. These two commonly used implant brands exhibit highly similar characteristics, with the most apparent difference being the variation in implant threading patterns. However, this distinction is not easily discernible for PA datasets.

Given the clinical importance of accurately identifying implant brands and the challenges posed by the subtle imaging differences between Biomet 3i and Xive implants on PAs, this study investigates whether these implants can be reliably distinguished by applying a deep learning-based model. We propose the following hypotheses:

**Null Hypothesis (H_0_)**. *The imaging characteristics of 3i and Xive implants are insufficiently distinct on PAs to enable reliable identification by newly trained clinicians and may result in a risk of misclassification even among experienced practitioners*.

**Alternative Hypothesis (H_1_)**. *Subtle but distinguishable imaging features exist between 3i and Xive implants on PAs, and these differences can be reliably detected using a deep learning-based classification model, improving the accuracy of implant brand identification compared to traditional clinical assessment*.

**Figure 2:**
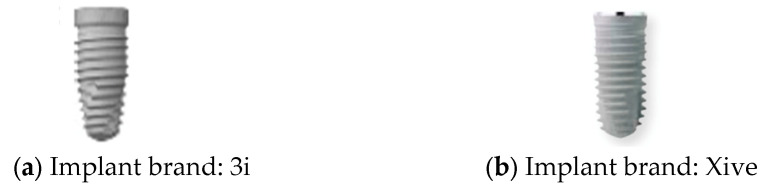
Schematic diagram of two implant brands.

Object classification is a highly relevant research topic in the medical field. Implant brand classification relies on manually labeled features to ensure interpretability in traditional methods. Dentists typically identify the 3i and Xive implant brands based on the shape of the implant platform. As shown in Figure 2, the platform of the 3i implant is generally wider, resulting in a more noticeable difference in thickness between the platform and the implant body. In contrast, the Xive implant has a platform nearly the same diameter as the body. Moreover, 3i and Xive have different types of dimension (D) and length (L), which are shown in Table 1. 3i implants are available in diameters ranging from 3.25 mm to 6 mm and lengths from 8.5 mm to 15 mm. In contrast, Xive implants offer diameters from 3.4 mm to 5.5 mm and lengths from 8 mm to 18 mm.

However, as previously mentioned, this approach poses a significant challenge for newly trained clinical dentists unfamiliar with various implant brands. Furthermore, for highly repetitive classification tasks, numerous AI-based solutions have been successfully applied in clinical diagnostics [23,24,25], demonstrating the growing trend of integrating deep learning with healthcare to enhance diagnostic efficiency. Deep learning object classification models applicable to PA can be broadly categorized into two primary types. The first type utilizes Convolutional Neural Networks (CNNs) for local feature extraction [26], with commonly used models including AlexNet, GoogleNet, Faster R-CNN, and YOLO. The second type leverages self-attention mechanisms for global feature extraction, employing transformer-based methods [27], such as Swin Transformer, ConvNeXt, and Vision Transformer. Deep learning-based approaches have been widely explored for PA analysis, with implants as primary evaluation targets. Several studies have demonstrated the effectiveness of CNN-based models in dental implant assessments. For instance, Zhang et al. [28] proposed a CNN model to predict implant failure, achieving an accuracy of 87%, enabling early clinical intervention for potential failures. Similarly, Chen et al. [29] employed two CNN models to detect implant locations and evaluate the extent of peri-implantitis-related damage. Additionally, Vera et al. [30] utilized YOLOv3 to assess peri-implantitis progression, demonstrating its capability in detecting marginal bone remodeling with a deep learning performance score of 0.898.

Despite these advancements, implant brand classification remains an underexplored area. As previously discussed, the accurate identification of implant brands is becoming increasingly essential in clinical dentistry. To address this gap, this study aims to develop a real-time implant brand detection system, facilitating faster and more accurate diagnoses while assisting newly trained dentists in their clinical decision-making process. Various image processing techniques are applied to improve image quality and enhance feature representation, ultimately increasing the accuracy of the final image recognition model and improving the precision of subsequent training. The YOLO model is utilized for training to identify the position of individual teeth and analyze implant brands. Finally, the model’s clinical evaluation time and classification accuracy are compared to ensure its robustness and reliability in real-world applications.

## 2. Materials and Methods

**Figure 3:**
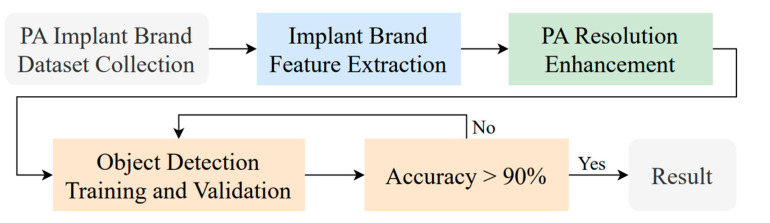
The main implant brand classification flowchart.

This section presents a detailed workflow for implant brand classification. Figure 3 illustrates the identification process of implant brands in PA used in this study. The process begins with collecting relevant implant brand images, which experienced dentists annotate with years of clinical expertise. Subsequently, the implant brand feature extraction technique and PA resolution enhancement method developed in this study are applied to improve object detection accuracy and visibility.

### 2.1. PA Implant Brand Dataset Collection

Currently, publicly available implant brand datasets are extremely limited. To our knowledge, no open-access PA dataset exists for implant brand classification. This study collected PA in collaboration with Chang Gung Memorial Hospital in Taoyuan, Taiwan, including 241 PAs from patients who underwent oral implant examinations between 2020 and 2023. The dataset was approved by the Institutional Review Board (IRB) of Chang Gung Medical Foundation (IRB No. 202301730B0). The inclusion criteria required that each PA contain at least one implant from the 3i or Xive brands. PAs from patients with a history of craniofacial abnormalities were excluded to avoid potential confounding factors. All eligible PAs that met the inclusion and exclusion criteria during the data collection period were consecutively included to maximize the sample size and ensure clinical representativeness. Implant brand data collection and ground truth annotation were conducted under the supervision of three oral specialists, each with over five years of clinical experience. The annotation process was performed manually using “LabelImg 1.8.1” software to ensure accurate implant localization. Each specialist independently annotated the implant brands without influencing one another, and the final brand assignment for each case was determined by majority voting to ensure annotation reliability.

**Figure 4:**
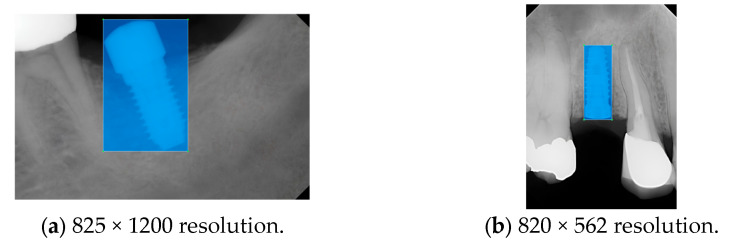
Example of an implant mask in PA.

Model training, validation, and testing procedures were conducted by trained research personnel under the supervision of senior investigators. A blinding protocol was implemented throughout the validation and testing phases to minimize operator bias. The operators performing model validation were blinded to the implant brand labels and the associated patient information during evaluation to ensure an objective assessment. Figure 4 illustrates the annotated PAs and their corresponding Ground Truth. Each implant brand was manually annotated using rectangular bounding boxes covering the entire visible region of the implant in the PA, including both the platform and the threaded structure. The imaging methodology and resolution details are shown in Table 2. The dataset consists of 241 PAs with image resolutions of either 825 × 1200 or 820 × 562 pixels. For experimentation, 20 images were set aside for testing, while the remaining images were split into a training set (75%) and a validation set (25%). The 75/25 training/validation split was chosen as a commonly used ratio in deep learning to ensure enough samples for training while retaining a representative validation set for performance monitoring [31,32].

### 2.2. Implant Brand Feature Extraction

**Figure 5:**
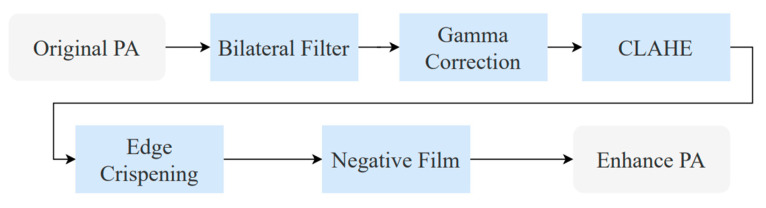
The optimal implant brand feature extraction flowchart.

PA provides a detailed view of the tooth apex and surrounding periodontal structures. This subsection proposes image enhancement methods to improve PA image detection accuracy and enable subsequent data comparison and cross-validation. The proposed methods and the optimal enhancement workflow are illustrated in Figure 5. The implant brand feature extraction method applies multiple image enhancement methods to improve implant brand detection accuracy and increase the model’s robustness.

#### 2.2.1. Bilateral Filter

**Figure 6:**
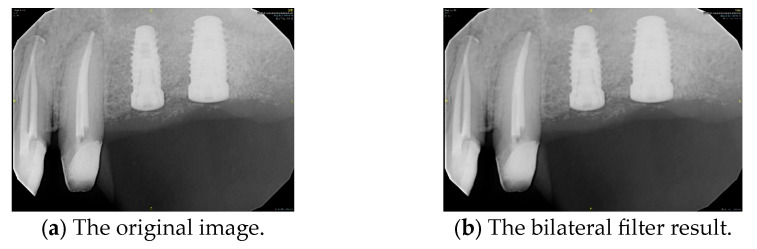
PA smoothed by the bilateral filter.

This study applies to the bilateral filter for PA smoothing, aiming to enhance image quality and reduce noise. The bilateral filter is a nonlinear filtering technique categorized as a frequency domain method. Unlike traditional image smoothing algorithms, the bilateral filter considers both the geometric proximity between pixels and differences in intensity and color, allowing it to reduce noise while preserving essential image details. In particular, preserving implant threads and platform shapes is crucial for distinguishing implant brands such as 3i and Xive, as these subtle features serve as key identifiers in clinical practice. We selected the bilateral filter over other smoothing methods because of its edge-preserving capabilities, critical for maintaining implant boundary features in PA. The goal is to perform a weighted averaging process within a local neighborhood for each pixel in PA, ensuring effective smoothing and improving the accuracy of subsequent image processing. The bilateral filter formulation [33] is shown in Equation (1) as follows.

(1) $$\vec{h}\left(\vec{x}\right)=k^{-1}\left(\vec{x}\right)\iint_{-\infty }^{\infty }\vec{f}\left(\vec{\xi }\right)c\left(\vec{\xi },\vec{x}\right)S\left(\vec{f}\left(\vec{\xi }\right),\vec{f}\left(\vec{x}\right)\right)d\vec{\xi }$$

where $\vec{h}$ represents the output image and $\vec{f}$ denotes the input image, with function values corresponding to the color information of a given pixel. Since the processed image may be a multi-channel color image rather than a single-channel grayscale image, both the input image $\vec{f}$ and the output image $\vec{h}$ re expressed as vectors. As a filter designed for image smoothing, the bilateral filter applies a weighted averaging process to each pixel within its local neighborhood, preserving image details while reducing noise. The filtering effect is illustrated in Figure 6.

#### 2.2.2. Gamma Correction

**Figure 7:**
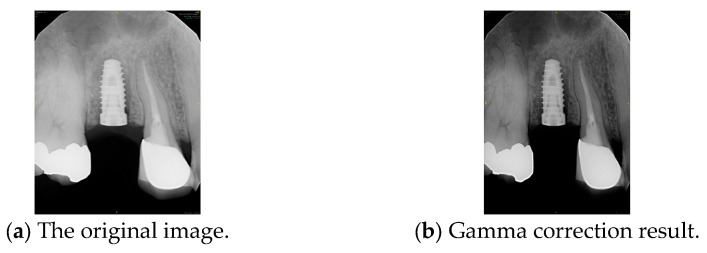
Comparison before and after gamma correction.

Gamma correction is applied to the following image processing: Gamma correction is a nonlinear operation used to adjust the luminance of light or tristimulus values in video and imaging systems. The gamma correction mathematical function [34] is given in Equation (2). Where A is a constant, input and output values are non-negative, real numbers. When the gamma value *γ* < 1, it is called encoding gamma, while *γ* > 1 is sometimes called decoding gamma. This study sets *γ* to 0.8 and A to 1, effectively enhancing low-intensity regions without saturating brighter areas. This encoding relationship is used to adjust brightness variations in an image. This study applies gamma correction to enhance the contrast between the implant and its surrounding structures, such as the gingiva and teeth. In other words, it makes the implant contours more distinct. As shown in Figure 7, gamma correction extracts the implant from the background.

(2) $$Vout=AV_{in}^{\gamma }$$

#### 2.2.3. Contrast-Limited Adaptive Histogram Equalization

**Figure 8:**
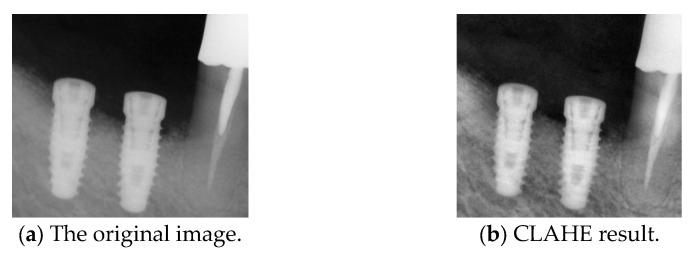
Comparison before and after CLAHE.

We applied an improved contrast-limited adaptive histogram equalization (CLAHE) technique, which enhances local contrast while preventing noise amplification caused by excessive enhancement. This method divides the image into multiple non-overlapping local regions and then independently applies histogram equalization to each grid. Compared to traditional Adaptive Histogram Equalization (AHE), CLAHE introduces a contrast limitation to prevent local oversaturation and minimize the effects of excessive enhancement. Additionally, CLAHE better controls noise levels, ensuring a balanced contrast adjustment. In this study, CLAHE was applied with a clip limit of 2.0 and a grid size of 8 × 8, which are commonly recommended settings for medical image enhancement [23,35]. Gamma correction is applied before CLAHE to optimize visibility further and adjust the brightness levels of the image. This combined approach enhances the separation between the implant and surrounding gingiva, improving implant detection accuracy, as shown in Figure 8.

#### 2.2.4. Edge Crispening

**Figure 9:**
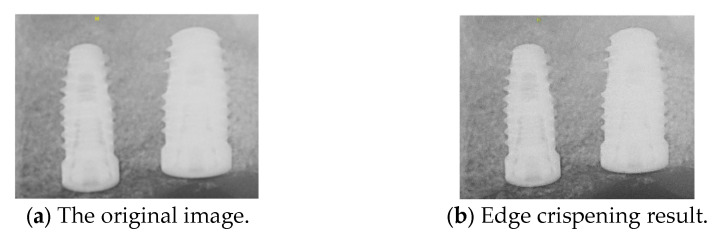
Comparison before and after edge crispening.

We apply the Laplacian filter to perform edge sharpening. The discrete Laplace operator is an analog of the continuous Laplace operator, defined to have meaning on a graph or a discrete grid. The discrete Laplacian operator is commonly used in image-processing applications. The discrete Laplacian operator is the sum of the second-order derivative Laplacian operator’s coordinate expressions. It is computed as the sum of the differences between the central pixel and its nearest neighbors. Since derivative filters are generally sensitive to image noise, a smoothing filter is typically applied before the Laplacian operator. However, using the Laplacian operator for edge crispening tends to introduce additional noise. Thus, we apply the bilateral filter for noise reduction before the Laplacian, and the comparison is shown in Figure 9. This approach enhances edge sharpening and reduces noise more effectively than applying edge sharpening alone.

#### 2.2.5. Negative Film

**Figure 10:**
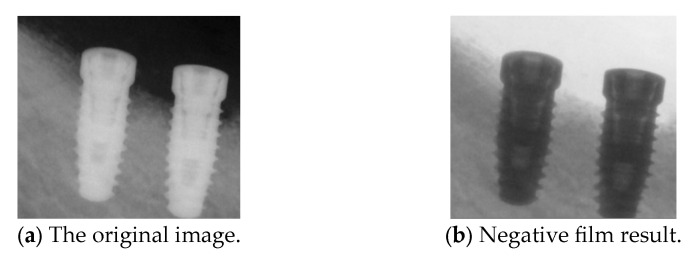
Comparison before and after the negative film.

Next, we apply negative films to enhance implant visualization. The purpose of the negative effect is to invert the original image’s colors and brightness, highlighting the fine details of the implant threading. This effect is achieved by subtracting the RGB pixel values of the target image from 255, effectively reversing the original colors of the regions. The use of this method in this study serves a dual purpose: it enhances the visibility of key details and acts as an effective data augmentation technique, increasing the diversity of the training dataset for improved model performance. The results of the negative effect are shown in Figure 10.

### 2.3. PA Resolution Enhancement

This subsection presents methods for enhancing PA resolution. The approach involves utilizing Dark Channel Prior for PA restoration and Lanczos interpolation for resolution enhancement, ensuring improved image clarity and detail preservation.

#### 2.3.1. Dark Channel Prior

Dark Channel Prior (DCP) is primarily used for image restoration and enhancement, assuming that in most natural images, at least one color channel in certain regions has very low-intensity values, often approaching zero, forming the “dark channel.” The gingiva is considered an unnecessary background in dental implant medical imaging analysis, while the implant is the primary target. By applying DCP, the implant can be effectively separated from the background, reducing the interference of the gingiva in implant detection. The DCP processing [36] is shown in Equations (3) and (4), where J_dark_(x) represents the dark channel value at each pixel x, J^c^(y) denotes the pixel intensity in the R, G, and B channels at pixel y, and Ω(x) is the local neighborhood centered around x. Based on the properties of the dark channel, in a clear (haze-free) image J(x), the dark channel value J_dark_(x) is usually minimal, ideally close to zero. Therefore, most studies estimate the transmission map It(x) using the dark channel of a hazy image I_dark_(x). Ω is an adjustment parameter, and A represents the global atmospheric light intensity.

(3) $$J_{dark}\left(x\right)={min}_{c\in \{R,G,B\}}({min}_{y\in \Omega \left(x\right)}\;\;J^{c}\left(y\right))$$

(4) $$It\left(x\right)=1-\omega \frac{J_{dark}\left(x\right)}{A}$$

**Figure 11:**
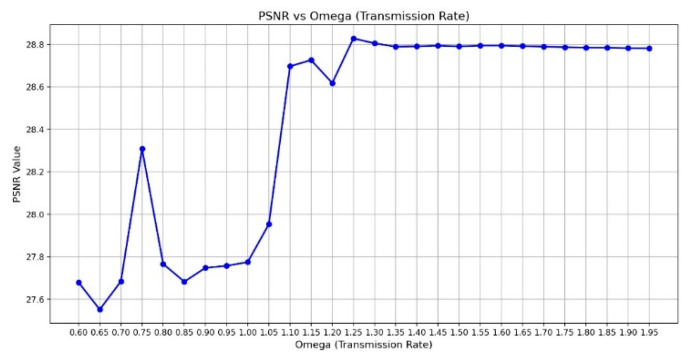
The chart of ω versus PSNR.

**Figure 12:**
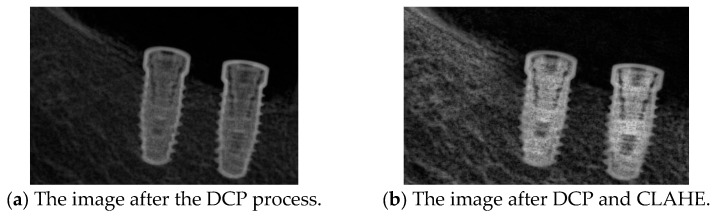
The DCP resolution result.

Peak Signal-to-Noise Ratio (PSNR) is computed using the standard formula based on the mean squared error (MSE) between the original and restored images. A higher PSNR value indicates better image reconstruction quality, and the PSNR [37] is shown in Equations (5) and (6), where m × n is the input image size, I(i,j) is the pixel intensity at position (i,j) in the original image, K(i,j) is the corresponding pixel intensity in the processed image, and MAX is the maximum possible pixel value of the image. The primary input parameter affecting the dehazing effect is the transmission map It(x). Figure 11 illustrates the relationship between ω and PSNR, which is analyzed to determine the optimal dehazing parameter. The results indicate that when ω is set to 1.25, the PSNR reaches its maximum value, representing the optimal dehazing condition. Based on this principle, the dazed image shown in Figure 12a is obtained. However, it can be observed that during the Dark Channel Prior computation, the local minimum operation within the neighborhood leads to the loss of fine details at the edges, resulting in partial implant details being removed. The CLAHE technique is applied to further enhance both the internal information and external contours of the implant, yielding the improved result shown in Figure 12b.

(5) $$MSE=\frac{1}{mn}\sum_{i=1}^{m}\sum_{j=1}^{n}{\left[I\left(i,j\right)-K(i,j)\right]}^{2}$$

(6) $$PSNR=10\log_{10}\left(\frac{{MAX}^{2}}{MSE}\right)$$

#### 2.3.2. Lanczos Interpolation

**Figure 13:**
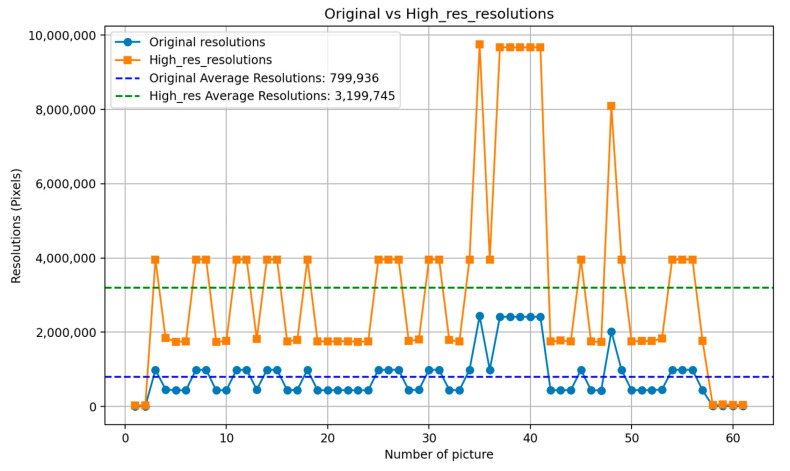
Comparison of the original image and the resolution enhanced by Lanczos interpolation.

Due to blurriness in some PAs, the implant contours may lack clarity, affecting the accuracy of implant detection and segmentation. This study applies Lanczos interpolation for image resampling, effectively enhancing image resolution and improving the visibility of delicate implant structures. Lanczos interpolation is a high-quality image resampling technique that utilizes Sinc function-based convolution operations to improve image resolution while minimizing aliasing and interpolation artifacts. Compared to bilinear or bicubic interpolation, the Lanczos interpolation technique primarily enhances implant threading contours and platform boundaries, which are subtle but critical features for distinguishing between 3i and Xive implant brands in PA. The mathematical formulation [38] of Lanczos interpolation is shown in Equations (7) and (8), where Sinc (x) =$\;\frac{\sin\left(\pi x\right)}{\pi x}$, and when x = 0, it is defined as 1. The parameter a represents the kernel window size, which is set to 2 in this study. The computation of each target pixel value I’(x,y) considers the weighted sum of surrounding original pixel values I(i,j). The resampling results shown in Figure 13 demonstrate that the resolution has been enhanced by approximately four times.

(7) $$L\left(x\right)=\left\{\begin{array}{l} \mathrm{sinc}\left(x\right)\cdot \mathrm{sinc}\left(\frac{x}{a}\right),\;\;\;\;\;\;\;\;\;\;\;\;\left|x\right|<a\\ 0,\;\;\;\;\;\;\;\;\;\;\;\;\;\;\;\;\;\;\;\;\;\;\;\;\;\;\;\;\;\;\;\;\;\;\;\mathrm{otherwise}\; \end{array}\right.$$

(8) $$I^{‘}\left(x,y\right)=\sum_{i=-a}^{a}\sum_{j=-a}^{a}I\left(i,j\right)\cdot L\left(x-i\right)\cdot L\left(y-i\right)$$

### 2.4. Object Detection Training and Validation

The proposed implant brand recognition system is based on an object detection model to locate the position of PA images. This study evaluates YOLOv8, YOLOv9, and YOLOv10, followed by implant localization. Compared to other state-of-the-art implant segmentation algorithms, the object detection-based approach reduces errors and execution failures caused by variations in PA imaging angles. The effectiveness of this approach will be further discussed in the Results section. Moreover, this study utilizes the computing platform detailed in Table 3 to train the YOLO models.

#### 2.4.1. YOLO Model

This study adopts a modified YOLOv10-based architecture to optimize implant detection and classification in PA, which is called the Implant Brand YOLOv10 detector (IB-YOLOv10), and the architecture is shown in Table 4. The proposed IB-YOLOv10 structure maintains the fundamental Backbone, Neck, and Head components while integrating enhanced feature extraction and detection optimization techniques. The key motivation behind these modifications was to improve the model’s ability to distinguish subtle morphological differences in implant threading and platform shapes, which are critical features for differentiating implant brands in PA. The Backbone leverages C2f modules and Spatial Pyramid Pooling-Fast (SPPF) to capture multi-scale implant features. The Neck applies feature fusion techniques, including upsampling and concatenation, ensuring improved object detection across different implant sizes. The Head is refined for precise classification and bounding box regression, effectively identifying implants more accurately. In this architecture, the image input will be normalized to 256 × 256 and use a 5 × 5 kernel size to sample the implant brand feature in the PA. Additionally, this study incorporates advanced post-processing techniques, including contrast enhancement, bilateral filtering, and gamma correction, to improve PA clarity and implant visibility. The optimized model enhances detection robustness and efficiency, ensuring reliable implant classification under varying imaging conditions.

#### 2.4.2. Experiment Setting

This study compares the performance of three different versions of the YOLO model. The hyperparameters of all three YOLO models were adjusted for fair performance comparison. The patience value was optimized to prevent premature training termination. However, excessive training may lead to overfitting; therefore, L2 regularization was applied to mitigate this issue. The hyperparameters used for the YOLO models are listed in Table 5. When training YOLO models, data augmentation hyperparameters are crucial in improving model performance. Among these, scale, translation, and mosaic augmentation are the most important, as they significantly enhance the model’s adaptability to various object sizes, positions, and backgrounds. Additionally, brightness, saturation, and mix-up augmentation further improve the model’s generalization ability.

#### 2.4.3. Validation Method

After training, we evaluated the model using four key metrics: accuracy, precision, recall, and F1-score. These metrics comprehensively assess the model’s predictive capability from multiple perspectives [23,24], including classification accuracy across different categories and overall performance in real-world applications, as shown in Equations (9)–(12). The evaluation is based on the confusion matrix, which consists of four components: true positive (TP), true negative (TN), false positive (FP), and false negative (FN). TP represents correctly predicted positive cases, while TN indicates correctly identified negative instances. FP occurs when a negative case is mistakenly classified as positive, and FN arises when a positive case is misclassified as negative. These values are the foundation for computing precision, recall, accuracy, and mAP50, providing insights into the model’s strengths and weaknesses. Extensive ablation studies and comparative experiments were conducted based on the image processing techniques and implant feature resolution enhancement methods described in the methodology section.

(9) $$Accuracy\;=\;\frac{TP+TN}{TP+TN+FP+FN}$$

(10) $$Precision=\frac{TP}{TP+FP}$$

(11) $$Recall=\frac{TP}{TP+FN}$$

(12) $$mAP50=\frac{1}{N}\sum_{i=1}^{N}AP_{i,50}$$

where $AP_{i,50}$ is the average precision.

## 3. Experiment Results

This section can be divided into two subsections. The first subsection explores how YOLO detects implants in PA images and compares it with recent studies on implant detection. The second subsection examines the effectiveness of our proposed implant brand feature extraction and PA resolution enhancement method in improving implant brand classification accuracy. Extensive ablation studies and comparative experiments demonstrated that our techniques offer significant advantages.

### 3.1. Original Implant Brand Dataset Training and Evaluation

In this subsection, the implant brand feature extraction and PA resolution enhancement methods are not applied. Instead, we evaluate PA-based implant detection and brand classification by adjusting the model and applying dataset augmentation. First, we consider training without dataset augmentation, and the results of the three YOLO architectures are shown in Table 6. The IB-YOLOv10 model achieved the highest overall precision of 56.8% for implant detection and brand classification, outperforming the other two YOLO models. However, other metrics were 2–4% lower than existing studies. For instance, YOLOv8 achieved the highest recall of 58.4%, whereas IB-YOLOv10 only reached 56.2% in our study. These results indicate that directly applying YOLO models for implant brand classification results in poor accuracy. Therefore, further dataset enhancement and image quality improvements are necessary to improve performance.

To further enhance model stability and classification accuracy, dataset augmentation techniques were incorporated into YOLO model training. These methods included Vertical Mirror Flip (VMF), Rotation of 15 Degrees (R15), and Gaussian Blur (GB). Gaussian Blur was explicitly applied to smooth implant threading features, simulating blurred PAs to evaluate YOLO’s robustness under such conditions. The original implant brand dataset consisted of 221 PAs, divided into 178 for training and 43 for validation. After applying image magnification (IM) through augmentation, the dataset size increased fourfold. According to the YOLO training results in Table 7, when comparing individual augmentation techniques, excluding Gaussian Blur resulted in higher accuracy. The highest accuracy among single augmentation methods was 67.8% when using Vertical Mirror Flip. Applying two or more augmentation techniques effectively mitigated the accuracy degradation and instability caused by image blurring. The highest accuracy for the two augmentation techniques was 74.7% using YOLOv10. When all three augmentation methods were applied, IB-YOLOv10 achieved the highest accuracy of 77.7%, whereas YOLOv8 had the lowest at 74.9%.

### 3.2. Enhancement Implant Brand Dataset Training and Evaluation

In this subsection, we explore the innovative implant brand feature extraction and PA resolution enhancement techniques developed in this study. The ablation experiment results are shown in Table 8, where the image enhancement techniques were broken down into multiple configurations. Bilateral filter (BF) and PA resolution enhancement (PARE) were considered mandatory components, while other enhancement techniques were tested separately for training and evaluation. The results showed that applying edge crispening (EC) and gamma correction (GC) improved the IB-YOLOv10 accuracy by at least 4%, achieving 81.2% and 82.6% accuracy. When both EC and GC were combined, the average precision increased to 89.9%. Further integrating the negative film effect (NFE) and CLAHE led to an 18% accuracy improvement over the baseline model without implant brand feature extraction and PA resolution enhancement, achieving a maximum precision of 95.5%. Moreover, no significant negative impact on model training was observed when applying any individual enhancement technique. However, it is worth noting that certain methods, such as BF and GC, provided only modest improvements when used alone. These findings suggest that although single methods may offer limited benefits, their combination produces a synergistic effect that enhances overall detection robustness. The integrated use of EC, GC, NFE, and CLAHE consistently improved performance across all evaluation metrics without introducing training instability, highlighting the advantage of a comprehensive enhancement strategy in implant brand classification tasks.

**Figure 14:**
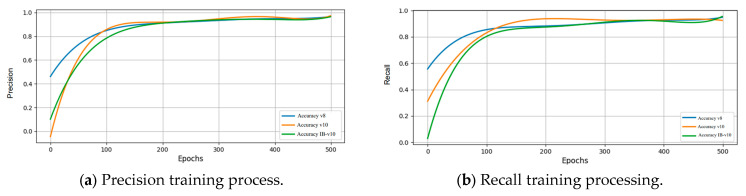
Different key metrics of training results after image enhancement.

Figure 14 illustrates the training process incorporating implant brand feature extraction and PA resolution enhancement techniques. The results show that precision and recall converge around 200 epochs, and the model is confirmed to have no overfitting issues at 500 epochs. The final IB-YOLOv10 model achieves approximately 95.3% precision and 96.8% recall, demonstrating model stability and the effectiveness of the image enhancement techniques to attain high-accuracy results.

Next, the optimal solution from the proposed methods was further evaluated for model stability, as shown in Table 9. We tested 40 images not included in the training or testing sets, and the IB-YOLOv10 model achieved an accuracy of 94.5%. Compared to YOLOv8, our model improved accuracy by 4.3%, and compared to YOLOv10, it achieved a 2.6% improvement. Table 10 presents the confusion matrix of IB-YOLOv10, showing that among 168 validation images, 95 3i implants and 70 Xive implants were correctly classified.

We selected 16 PA (eight from each of the two resolution types) to evaluate the IB-YOLOv10 model for validation. The validation results are presented in Table 11, showing that the classification accuracy for both 3i and Xive implants exceeds 90% across different resolutions, with accuracy and recall. Additionally, we compared the implant brand identification time required by our model against that of senior dentists. Three senior dentists with over five years of clinical experience independently performed implant brand identification on PA. To ensure fairness and consistency, each dentist evaluated without access to any implant databases and without discussing their decisions with others. This protocol was designed to prevent bias and maintain the reliability of the comparison. The results indicate that IB-YOLOv10 achieves an average inference time of 6.47 ms, whereas a senior dentist requires 4.58 s for manual identification. This demonstrates that IB-YOLOv10 can significantly reduce the time necessary for implant brand classification, thereby minimizing the diagnostic workload in daily clinical practice.

## 4. Discussion

The primary objective of this study is to apply deep learning techniques to alleviate the challenges and workload faced by new dentists in clinical implant brand classification. This study used PA as the imaging dataset and implemented a series of implant brand feature extraction and resolution enhancement techniques. Furthermore, this study proposed an improved YOLOv10-based implant brand detection model called IB-YOLOv10. Three key innovations and contributions of this paper are as follows:

1. We are the first study to propose a deep learning-based approach for 3i and Xive implant brand detection in PA, aiming to assist new and experienced dentists in clinical diagnosis. Our proposed method achieves 94.5% accuracy in 3i and Xive implant brands across two common PA resolutions in real-world clinical scenarios.
2. This study proposes IB-YOLOv10, an object detection model for implant brand classification based on YOLOv10. Compared to YOLOv8 and YOLOv10, IB-YOLOv10 improves detection accuracy by 4.3% and 2.6%.
3. This study introduces a novel feature extraction method for implant brand classification by integrating multiple image processing techniques and a resolution enhancement technique based on Lanczos interpolation and Dark Channel Prior. The experimental results show that, compared to the original dataset, applying implant brand feature extraction and PA resolution enhancement in IB-YOLOv10 increases implant brand detection accuracy by 17.8%.

Moreover, we compared the performance of our IB-YOLOv10 model with existing implant detection techniques applied to PAs. Table 12 shows that our model achieves the highest accuracy (94.5%), precision (93.8%), and mAP50 (99.2%) among the studies compared. In terms of accuracy, IB-YOLOv10 outperforms Lee et al. [39] by 2.3%, Park et al. [40] by 10.7%, and Lee et al. [41] by 3.9%, indicating a consistent improvement in correct implant disease identification. Regarding precision, our model demonstrates a substantial advantage over Lee et al. [39] (80.0%) and Park et al. [40] (81.5%) and achieves a comparable performance to Lee et al. [41] (92.0%). This suggests that IB-YOLOv10 can effectively minimize false identifications, critical in reducing unnecessary clinical interventions. For mAP50, our model achieved 99.2%, outperforming Park et al. [40], who reported 83.8%. However, in terms of recall, IB-YOLOv10 (93.3%) is slightly lower than Lee et al. [39] (94.7%) and Lee et al. [41] (92.0%). The marginally lower recall of our model may be attributed to the narrower detection focus on implant disease compared to broader detection scopes in other studies.

Our proposed IB-YOLOv10 model demonstrates a comparable level of precision to existing implant identification models, indicating similar accuracy in predictions with a controlled rate of false positives. Nevertheless, our model expands its applicability to the classification of two commonly used implant brands, Biomet 3i and Xive, which enhances its relevance in clinical dentistry. This improvement is especially valuable in dental practice, as it reduces misclassification risk and minimizes the diagnostic workload for clinicians, thereby making implant brand identification more reliable, scalable, and clinically practical. However, this study still has some physical limitations. The first limitation is the restricted availability of the implant brand dataset. Since PA involves patient privacy, its use in research requires patient consent and IRB approval. Since implant brand classification has become an essential aspect of modern clinical practice, future work will focus on obtaining more patient consent and collaborating with multiple medical clinics to build a more diverse PA dataset. This will enhance the model’s accuracy, stability, and robustness. The implant feature extraction method suffers from excessive background noise, obscuring the implant and hindering accurate detection. Future research will explore alternative image processing techniques to filter out background regions effectively. The third limitation is that the required memory size for model deployment in medical institutions could become a significant burden with a larger image dataset. Future research will explore model quantization and pruning techniques to accelerate model inference while maintaining detection accuracy and helping meet the real-time clinical requirements of dentists.

## 5. Conclusions

IB-YOLOv10 provides new and experienced dentists with a highly convenient implant brand detection assistance model. By leveraging deep learning, the model aids dentists in determining the implant brand while improving accuracy through feature extraction and resolution enhancement techniques. This model offers dentists a fast and effective auxiliary tool, providing a better solution for competent dental healthcare.

## Figures and Tables

**Table 1:** Specifications of implant dimensions and lengths for the 3i and Xive brands.

Implant Brand	3i	Xive
Dimension (D)	3.25 mm/4 mm/5 mm/6 mm	3.4 mm/3.8 mm/4.5 mm/5.5 mm
Length (L)	8.5 mm/10 mm/11.5 mm/13 mm/15 mm	8 mm/9.5 mm/11 mm/13 mm/15 mm/18 mm

**Table 2:** Implant brands collected in the dataset and the PA imaging methodology.

PA Imaging Methodology			
Exposure Time	Incrementally adjustable from ≤ 0.03 to 3.2 s	Image developing speed	≤5 s
X-Ray Generator	High frequency generator for constant high	Sensor size (mm)	31.3 × 44.5
Image Size	825 × 1200 or 820 × 562	Image format	DCI
The dataset includes two implant brands: 3i and Xive			
3i	164	Xive	77
Training Set	Validation Set	Test Set	Total
178	43	20	241

**Table 3:** The hardware and software platform version.

Hardware Platform	Version
CPU	AMD Ryzen™ R7-7700@3.80 GHz
GPU	NVIDIA GeForce RTX 3070 8G
DRAM	64 GB
Software platform	version
Python	3.9.31
PyTorch	2.4 + cu121
CUDA	12.1

**Table 4:** IB-YOLOv10 architecture.

Layer	Layer Type	Kernel Size	Stride	Filters Number	Future Map Size
1	Convolution	5 × 5	2	64	256 × 256 × 16
2	Convolution	3 × 3	2	128	128 × 128 × 32
3	C2f	-	-	256	128 × 128 × 64
4	Convolution	3 × 3	2	256	64 × 64 × 64
5	C2f	-	-	512	64 × 64 × 128
6	Convolution	3 × 3	2	512	32 × 32 × 128
7	C2f	-	-	512	32 × 32 × 128
8	Convolution	3 × 3	2	1024	16 × 16 × 256
9	C2f	-	-	1024	16 × 16 × 256
10	SPPF	5 × 5	-	1024	16 × 16 × 256
11	C2PSA	-	-	1024	16 × 16 × 256
12	Upsample	-	-	1024	32 × 32 × 256
13	Concatenation	-	-	1024	32 × 32 × 256
14	C2f	-	-	512	32 × 32 × 128
15	Upsample	-	-	512	64 × 64 × 128
16	Concatenation	-	-	512	64 × 64 × 128
17	C2f	-	-	256	64 × 64 × 64
18	Convolution	3 × 3	2	256	32 × 32 × 128
19	Concatenation	-	-	256	32 × 32 × 128
20	C2f	-	-	512	32 × 32 × 128
21	Convolution	3 × 3	2	512	16 × 16 × 128
22	Concatenation			512	16 × 16 × 128
23	C2f			20 × 20 × 256	16 × 16 × 128

**Table 5:** YOLO model hyperparameter settings.

Hyperparameter	Value	Hyperparameter	Value
Initial Learning Rate	0.0005	Hue	0.015
Final Learning Rate	0.1	Saturation	0.7
Image Size	256	Brightness	0.4
Epochs	500	Translation	0.1
Batch	16	Scale	0.5
Stopping Patience	50	Horizontal flip	0.5
L2 Regularization	0.0007	Mosaic augmentation	0.8
Momentum	0.937	Mixup augmentation	0.2

**Table 6:** The original YOLO model training result.

	Accuracy	Recall (Average)	Recall (Max)	Precision (Average)	Precision (Max)	mAP50 (Average)	mAP50 (Max)
YOLOv8	50.6%	56.3%	58.4%	58.2%	59.9%	58.9%	58.4%
YOLOv10	53.8%	55.9%	57.6%	54.8%	57.7%	59.1%	57.7%
IB-YOLOv10	56.8%	52.2%	56.2%	54%	57.3%	55.2%	56.8%

**Table 7:** Implant brand PA dataset augmentation and training result.

VMF	R15	GB	IM	Accuracy (8, 10, IB-v10)	mAP50 (8, 10, IB-v10)	Precision (8, 10, IB-v10)	Recall (8, 10, IB-v10)
			1	50.6/53.8/56.8	58.4/57.7/56.8	59.9/57.7/57.3	58.4/57.6/56.2
✓			2	57.6/66.1/67.8	52.9/53.2/60.5	53.1/61.9/67.0	55.4/59.9/65.9
	✓		2	60.4/66.2/66.6	57.5/55.5/59.8	64.7/61.6/65.1	64.1/63.2/57.8
		✓	2	65.0/64.2/64.8	57.3/60.2/61.8	55.3/61.5/57.7	53.8/60.2/58.4
✓	✓		3	71.4/73.4/72.1	68.3/71.6/72.9	68.8/74.5/72.4	74.9/75.2/71.8
	✓	✓	3	71.5/74.7/73.3	68.2/72.3/71.5	71.2/70.4/73.3	75.6/73.7/78.3
✓		✓	3	71.6/69.6/72.4	68.6/69.3/74.1	70.7/71.3/74.2	71.2/74.2/76.3
✓	✓	✓	4	74.9/75.7/77.7	73.4/74.5/76.3	76.9/74.1/77.5	71.2/73.4/78.7

**Table 8:** Training accuracy evaluation with implant brand feature extraction and PA resolution enhancement.

BF	EC	GC	CLAHE	NFE	PARE	Accuracy (8, 10, IB-v10)	mAP50 (8, 10, IB-v10)	Precision (8, 10, IB-v10)	Recall (8, 10, IB-v10)
						74.9/75.7/77.7	73.4/74.5/76.3	76.9/74.1/77.5	71.2/73.4/78.7
✓	✓				✓	77.3/78.1/81.2	78.6/83.0/82.5	78.7/82.4/81.1	79.3/80.5/81.6
✓		✓			✓	81.8/79.5/82.6	76.6/83.4/83.7	82.3/84.5/83.8	81.2/82.3/83.4
✓	✓	✓			✓	84.6/87.2/89.9	80.2/88.6/90.2	88.2/91.6/92.1	88.9/91.7/91.6
✓	✓	✓	✓		✓	86.7/91.5/93.4	84.7/90.3/91.0	88.2/91.5/93.9	88.6/91.8/92.1
✓	✓	✓	✓	✓	✓	90.8/93.3/95.5	93.5/93.7/96.7	91.9/93.5/96.8	92.0/93.2/95.3

BF = bilateral filter, EC = edge crispening, GC = gamma correction, CLAHE = contrast-limited adaptive histogram equalization, NFE = negative film effect, PARE = PA resolution enhancement.

**Table 9:** Accuracy evaluation of a randomly selected test set of 40 PA.

	Accuracy (Average)	Recall (Average)	Recall (Max)	Precision (Average)	Precision (Max)	mAP50 (Average)	mAP50 (Max)
YOLOv8	90.2%	92%	96.9%	91.9%	98.3%	92.9%	94.2%
YOLOv10	91.9%	93.2%	98.4%	93.5%	98.1%	93.2%	96.7%
IB-YOLOv10	94.5%	93.3%	99.9%	96.8%	99.2%	96.4%	98.2%

**Table 10:** IB-YOLOv10 confusion matrix.

		Actual	
		3i	Xive
Predicted	3i	95	1
	Xive	2	70

**Table 11:** Implant brand detection results across different image resolutions.

Image Resolution = 825 × 1200				
Test Image 1–4	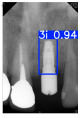	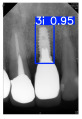	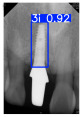	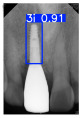
Accuracy	94.13%	95.29%	92.88%	91.80%
Recall	96.71%	94.03%	93.88%	92.15%
Model reference time	6.57 ms	7.08 ms	7.12 ms	6.43 ms
Dentists’ average diagnostic time	2.78 s	4.55 s	7.78 s	7.23 s
Test Image 5–8	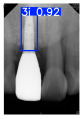	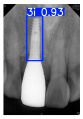	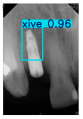	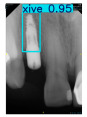
Accuracy	92.44%	93.38%	96.61%	95.89%
Recall	94.60%	96.85%	95.22%	95.49%
Model reference time	6.99 ms	7.34 ms	6.11 ms	6.76 ms
Dentists’ average diagnostic time	5.79 s	3.87 s	4.11 s	2.45 s
Image resolution = 820 × 552				
Test Image 9–12	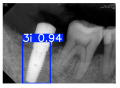	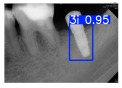	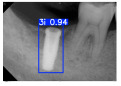	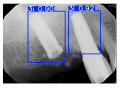
Accuracy	94.11%	95.06%	94.82%	90.97%/92.86%
Recall	96.19%	95.72%	95.99%	94.12%/92.75%
Model reference time	6.18 ms	6.12 ms	6.48 ms	5.57 ms
Dentists’ average diagnostic time	2.51 s	5.65 s	4.51 s	3.44 s
Test Image 13–16	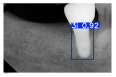	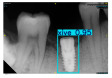	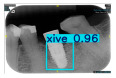	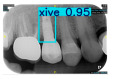
Accuracy	92.03%	95.74%	96.79%	95.17%
Recall	93.59%	94.97%	94.13%	95.72%
Model reference time	6.45 ms	6.07 ms	6.01 ms	6.22 ms
Dentists’ average diagnostic time	3.47 s	6.89 s	3.45 s	4.77 s

**Table 12:** Comparison of implant detection results with other studies.

	Accuracy	Recall	Precision	mAP50
This study	94.5%	93.3%	93.8%	99.2%
Lee et al. [39]	92.2%	94.7%	80.0%	×
Park et al. [40]	83.8%	86.0%	81.5%	83.8%
Lee et al. [41]	90.6%	92.0%	92.0%	×

## Data Availability

The data used in this study are confidential and cannot be provided to any external parties.
